# Identification of novel Lynch syndrome mutations in Chinese patients with endometriod endometrial cancer

**DOI:** 10.20892/j.issn.2095-3941.2019.0295

**Published:** 2020-05-15

**Authors:** Caixia Ren, Yan Liu, Yuxiang Wang, Yan Tang, Yawei Wei, Congrong Liu, Hongquan Zhang

**Affiliations:** ^1^Key Laboratory of Carcinogenesis and Translational Research, Ministry of Education, and State Key Laboratory of Natural and Biomimetic Drugs, Peking University Health Science Center, Beijing 100191, China; ^2^Department of Human Anatomy, Histology and Embryology, Peking University Health Science Center, Beijing 100191, China; ^3^Department of Pathology, Peking University Health Science Center, Beijing 100191, China; ^4^Department of Human Anatomy, Basic Medical College of Hebei North University, Zhangjiakou 075000, China

**Keywords:** DNA mismatch repair, endometrial endometrioid cancer, germline mutation, Lynch syndrome, next-generation sequencing

## Abstract

**Objective:** Lynch syndrome (LS) predisposes patients to early onset endometrioid endometrial cancer (EEC). However, little is known about LS-related EEC in the Chinese population. The aim of this study was to investigate the prevalence of LS and to identify the specific variants of LS in Chinese patients with EEC.

**Methods:** We applied universal immunohistochemistry screening to detect the expression of mismatch repair (MMR) proteins, which was followed by MLH1 methylation analysis to identify suspected LS cases, next-generation sequencing (NGS) to confirm LS, and microsatellite instability (MSI) analysis to verify LS.

**Results:** We collected 211 samples with EEC. Twenty-seven (27/211, 12.8%) EEC cases had a loss of MMR protein expression. After MLH1 methylation analysis, 16 EEC cases were suggested to be associated with LS. Finally, through NGS and MSI analysis, we determined that 10 EEC (10/209, 4.78%) cases were associated with LS. Among those cases, 3 unreported mutations (1 frameshift and 2 nonsense) were identified. *MSH6* c.597_597delC, found in 4 patients, is likely to be a founder mutation in China.

**Conclusions:** We demonstrated the feasibility of a process for LS screening in Chinese patients with EEC, by using universal immunohistochemistry screening followed by MLH1 methylation analysis and confirmation through NGS and MSI analysis. The novel mutations identified in this study expand knowledge of LS.

## Introduction

Lynch syndrome (LS) is an autosomal dominant inherited cancer susceptibility syndrome that predisposes people to early-onset colorectal cancer (CRC) and other associated cancers, particularly endometrioid endometrial cancer (EC). LS is caused by a genetic mutation in one of several DNA mismatch repair (MMR) genes: *MLH1*,* MSH2*,* MSH*6, or *PMS2*^1^. The estimated risk of CRC in women with LS is 39%–54%. However, the cumulative lifetime risk of endometrial cancer for these individuals is 50%–60%. Thus gynecologic cancers act as sentinel cancers in women^2,3^. Identification of LS either after diagnosis with a sentinel cancer or in the premalignant phase is critical to provide patients and their family members with an opportunity for surveillance of other LS-associated cancers, especially CRC; such surveillance can decrease CRC mortality by more than 60%^4,5^ and can also eliminate unnecessary fear and intensified surveillance for unaffected relatives.

Although its adoption has been slow for almost a decade, screening for LS-associated gynecologic cancers has recently been brought into clinical practice. Current data indicate that the prevalence of LS in various patient populations with gynecologic cancer varies^6^. LS-associated ECs are primarily endometrioid EC (EEC), although non-endometrioid subtypes, including clear cell, papillary serous, undifferentiated carcinoma, and carcinosarcoma, have been reported^7–10^. Few studies on LS-associated EEC in the Chinese population have been published. Comprehensive sequencing analysis of germline DNA has also been scarce.

Next-generation sequencing (NGS) approaches, as compared with traditional single-gene Sanger sequencing, have revolutionized the speed, throughput, and cost-effectiveness of DNA sequencing^11,12^. In this study, we propose a selective screening process for LS in Chinese patients with EEC by using immunohistochemistry (IHC) followed by MLH1 methylation analysis, and further confirmation by NGS and microsatellite instability (MSI) analysis. We used NGS to sequence all exons, including the splice junctions of MMR genes, to identify specific mutations in the Chinese population.

## Materials and methods

### Patients

This study was approved by the Peking University Institutional Review Board, Beijing, China. All methods were performed in accordance with the relevant guidelines and regulations. All patient-derived samples were collected after verbal informed consent was obtained (Supp_Mat1).

A total of 211 EECs were collected without age restriction at the time of hysterectomy, oophorectomy, and biopsy in the Peking University Third Hospital. Cases were selected in a population-based consecutive series between March 2000 and August 2015 according to the availability of pathologic materials for analysis. Hematoxylin and eosin stained slides of formalin-fixed paraffin embedded (FFPE) tissues were retrieved from the surgical pathology files and reviewed; pathological diagnoses were then confirmed, and appropriate tumor and normal tissue blocks were selected for study by a gynecologic pathologist. Cases were not included if too little tumor tissue was available for analysis. Tumor-infiltrating lymphocytes were considered to be increased when they equaled or exceeded 40/10 high-powered fields. Peritumoral lymphocytes (Crohn’s-like lymphocytic infiltrate) were observed only in hysterectomy samples. Tumor location was assigned on the basis of the gross descriptions recorded in the pathology reports as well as histologic assessments for the lower uterine segment. Family history data were collected from medical archives and in follow-up.

### IHC

Sections of the selected paraffin embedded tissue blocks from all 211 patients were immunostained for the MMR proteins MLH1, MSH2, and MSH6. IHC was performed as described previously^13^. In brief, 4 μm thick FFPE tissue sections were stained with primary monoclonal rabbit antibodies to MSH2 (Origene, USA, ZA-0622), MSH6 (Origene, USA, ZA-0541), and MLH1 (Origene, USA, ZM-0154) with an EnVision + Dual Link System (K4061; Dako). Microwave antigen recovery with citrate buffer (pH 6.0 for MSH2 and MSH6) or Tris-EDTA buffer (pH 9.0 for MLH1) was performed. Diaminobenzidine was used as the chromogen, and hematoxylin was used as the counterstain. Negative control reactions used Tris-buffered saline instead of the specific primary antibody, and no positive staining was observed. Normal expression of protein was defined by the presence of nuclear staining in EC cells. Loss of staining in carcinoma with concurrent positive staining in nuclei of normal endometrial epithelial cells indicated an absence of protein expression. In addition to normal tissue slices, adjacent normal stroma and lymphocytes within cancer specimens served as internal controls for proper staining in each case. The results were considered unreliable when no immunostaining of normal tissue was observed. The processed IHC slides were blindly evaluated by 2 pathologists.

### MLH1 methylation analysis

For cases with a loss of MLH1 protein expression, PCR based MLH1 promoter methylation analysis was performed. DNA was isolated from FFPE tissue sections that were microdissected with a scalpel blade to provide relatively pure tumor samples for analysis. Isolated DNA was treated with bisulfite with a Qiagen Epitect Kit (Qiagen; Valencia, CA) to convert methylated cytosine to uracil. For each tumor DNA sample, 2 separate PCRs were used to amplify methylated (M) and unmethylated (U) *MLH1* gene promoters (MLH1-M forward, 5′-ACGTAGACGTTTTATTAGGGTCGC-3′ and *MLH1*-M reverse, 5′-CCTCATCGTAACTACCCGCG-3′; *MLH1*-U forward, 5′-TTTTGATGTAGATGTTTTATTAGGGTTGT-3′ and *MLH1*-U reverse, 5′-ACCACCTCATCATAACTACCCACA-3′). PCR products were run on 2% agarose gels and visualized under ultraviolet light. The RKO colon carcinoma cell line was used as a positive control because it is known to have a loss of MLH1 protein due to *MLH1* promoter methylation; the leukemia cell line K562 was used as a negative control with no* MLH1* methylation.

### Germline mutation analysis (NGS)

Patients with suspected LS were candidates for NGS. Suspicion of LS was based on IHC and the MLH1 methylation status. MLH1 genetic testing was performed in cases in which tumors showed a loss of protein expression and unmethylated MLH1. Total genomic DNA was extracted from normal FFPE tissues including lymph nodes and oviducts with a commercially available DNA extraction kit, GeneRead DNA FFPE Kit (Qiagen, Hilden, Germany). The quantity and quality of the DNA samples was determined with a Qubit 2.0 fluorometer (Invitrogen, Carlsbad, CA, USA).

All exons of the *MLH1*, *MSH2*, and *MSH6* genes, including splice junctions, were screened with Ion Torrent semiconductor sequencing. Primers of amplicons covering the CDS region and flanking regulation sequences of each targeted gene were automated and designed with the Ion AmpliSeq™ Ready-to-Use custom designer platform (https://www.ampliseq.com/protected/dashboard.action). Ultrahigh-multiplex PCRs were performed in one tube in parallel, and the primers were mixed and provided in 2 primer pools. Eventually, 97.09% of the 12.59 kb targeted region was overlapped by 129 amplicons 125–175 bp in length. Ion Torrent adapter-ligated libraries were built with an Ion AmpliSeq™ Library Kit 2.0 (Life Technologies) according to the manufacturer’s protocol. Raw data were initially processed in the Ion Torrent platform-specific software Torrent Suite v4.6 to generate sequence reads, trim adapter sequences, align sequences to the hg19 human reference genome, analyze coverage, and call variants. All variants were then handled with the online bioinformatic software Ion Reporter 5.0 (https://ionreporter.thermofisher.com/ir). Variants in the current study were filtered out and were not included in further evaluations, such as the 5′ and 3′ untranslated regions, coverage < 100×, and variant allele frequency < 10%. The mean depth of coverage was 294× (range 187–451), and the mean on-target percentage was 93.45%.

All sequence variant descriptions were verified with VariantValidator (https://variantvalidator.org/). Mutations leading to a truncated or unstable protein are considered clearly pathogenic and are diagnostic of LS. These mutations include nonsense, frameshift, and splice site mutations^7^. To determine their pathogenicity, all mutations were checked against 3 well-established and relevant databases: the LOVD database maintained by the International Society for Gastrointestinal Hereditary Tumours (InSiGHT, www.insight-group.org), the Human Gene Mutation Database (www.hgmd.cf.ac.uk/ac/index.php), and the National Center for Biotechnology Information Search database (http://www.ncbi.nlm.nih.gov/). A missense variant was considered pathogenic only if it was classified as pathogenic or disease causing by these databases. The functional effects of missense mutations unreported in these databases were predicted with PolyPhen-2 (http://genetics.bwh.harvard.edu/pph2/) and Sift (http://sift.jcvi.org/) to determine likely pathogenic mutations. Detailed information is shown in Supp_Mat2.

### MSI analysis

MSI Analysis System Version 1.2(^a-e^) was used to detect MSI in the samples detected as NGS positive. The MSI Analysis System included fluorescently labeled primers for co-amplification of 7 markers including 5 mononucleotide repeat markers (BAT-25, BAT-26, NR-21, NR-24, and MONO-27) and 2 pentanucleotide repeat markers (Penta C and Penta D). Mononucleotide markers were used for MSI determination, and pentanucleotide markers were used to detect potential sample mix-ups or contamination. An internal lane size standard was added to the amplified samples to ensure accurate sizing of alleles and to adjust for run-to-run variation. The PCR products were separated by capillary electrophoresis with an ABI PRISMR 310 or 3100 or Applied BiosystemsR 3130 or 3130*xl* Genetic Analyzer, and the output data were analyzed with GeneMapperR software (Applied Biosystems) to determine the MSI status of test samples. To simplify data analysis, we created panels and bins text files to enabled automatic assignment of genotypes in GeneMapperR software. Samples in which ≥40% of microsatellite markers were altered (≥2 altered markers out of 5) were classified as MSI-High (MSI-H).

The cycling profile was as follows: 95 °C for 11 min; 96 °C for 1 min; 94 °C for 30 s, ramp 68 s to 58 °C, hold for 30 s, ramp 50 s to 70 °C, and hold for 1 min for 10 cycles; 90 °C for 30 s, ramp 60 s to 58 °C, hold for 30 s, ramp 50 s to 70 °C, and hold for 1 min for 20 cycles; 60 °C for 30 min; and 4 °C hold.

### Statistical analysis

All statistical analyses were performed in SPSS statistical software (version 15.0). The chi-squared test was used to compare qualitative variables. The age between groups was compared with *t* tests. The significance level was set at *p* < 0.05.

## Results

### IHC analysis of tumor tissues

**Figure 1:**
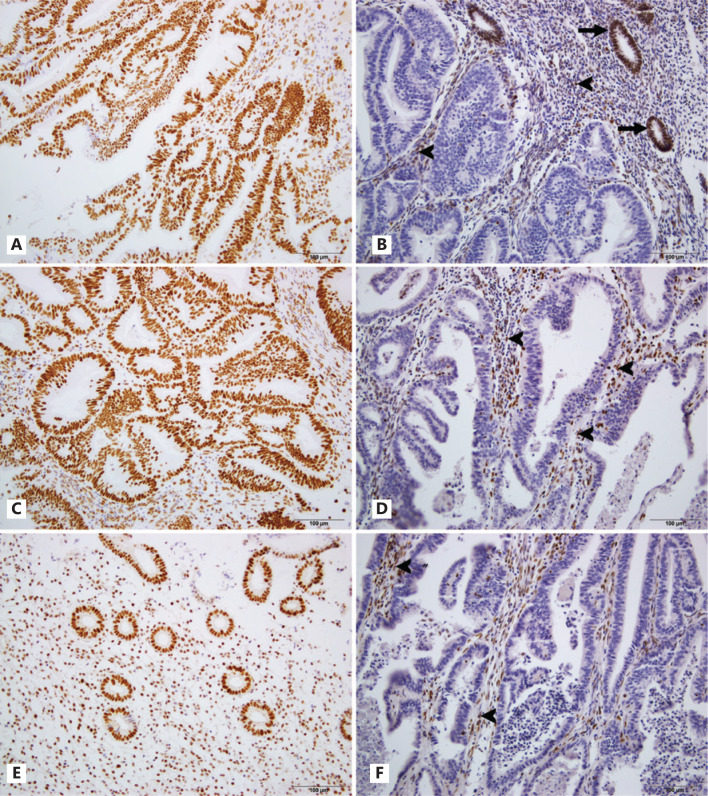
Expression of MMR protein in EEC. A. MSH2 retained in EEC; B. MSH2 was deficient in EEC; C. MSH6 expressed in EEC; D. Loss of MSH6 in EEC; E. MLH1 retained in endometrial cells; F. MLH1 was lost in EEC. Normal stroma cells (arrowheads) and endometrial cells (arrows) served as internal positive control.

We immunostained 211 EEC samples. The mean age of the patients with EEC was 54.64 years (**Table 1**). Of the EEC samples examined, 12.8% (27/211) had lost the expression of at least 1 MMR protein (MMR deficiency) (**Figure 1, Table 1**).

Among 27 cases of MMR-deficient EEC (mean age: 56.22 years), 13 (48.15%) were characterized by MLH1 loss (mean age: 60 years), 10 (37.04%) were characterized by MSH2/MSH6 loss (mean age: 51.3 years), and 4 (14.81%) were characterized by solitary MSH6 loss (mean age: 56.25 years) (**Table 2**). There were no differences in clinicopathologic characteristics between the MMR-deficient EEC and MMR-intact EEC cases (**Table S1**).

### Methylation analysis of MLH1

Among the EEC samples deficient in MLH1 expression, 69.23% (9/13) had MLH1 promoter methylation, and the remaining MLH1-deficient EEC samples (4/13) that lacked MLH1 methylation were designated as having presumed LS (**Figure S1**). We did not detect statistically significant differences in the clinicopathologic variables in these groups (**Table S2**).

### NGS analysis of MMR

Among 18 suspected LS-associated EEC cases, 2 samples were excluded because of sample quality, and only 16 EEC samples were analyzed by NGS. Pathogenic or likely pathogenic mutations were found in 10 (62.5%, 10/16) suspected LS-associated EEC samples, which represented 4.78% (10/209) of the overall EEC cohort. Among these, 7 cases with a *MSH6* mutation, 2 with a *MSH2* mutation, and 1 with a *MLH1* mutation were detected. The mean age of all mutation carriers was 52.2 years (**Table 3**). The MMR gene mutation types detected in this study are shown in **Table 4**.

Two frameshift mutations were found in *MSH6*. One of them, detected in 4 unrelated patients, was caused by the homozygous deletion of a cytosine at the nucleotide position c.597 (c.597_597delC), which leads to p.Ser200fs and produces a premature stop at codon 210 (TAG). The variant allele frequency of this mutation was 100%. The clinical phenotypes of 4 patients with c.597_597delC in our study are shown in **Table 5**. The International Federation of Gynaecology and Obstetrics (FIGO) stage of these EECs was I–II, and the histological grade was G2–G3. These patients’ mothers and their mothers’ siblings or their own siblings developed various cancers. Patient End 001 (50 years old), whose mother developed cervical adenocarcinoma, had a brother with CRC, another brother with lung cancer, an uncle with CRC, and another uncle with liver cancer. Patient End 002 was 60 years old. Her mother had cervical adenocarcinoma, and her brother had CRC. Patient End 003 was 66 years old. Her mother had cervical adenocarcinoma. Patient End 004 (49 years old) developed colorectal adenomas 10 years after EEC onset. Her mother had CRC. Except for the probands, all cancer patients had died, and their offspring had not developed cancers at the time of the study.

The other *MSH6* frameshift mutation was due to the insertion of GCAAG at c.3906_3907 (c.3906_3907insGCAAG), thus leading to p.Leu1305fs and producing a premature stop at codon 1328 (TGA). This mutation was not reported in any database.

There were 2 unreported nonsense mutations, c.71C > A p.Ser24Ter in *MSH6* and c.2092G > T p.Glu 698Ter in *MSH*2, which resulted in premature stop sequences at codons 24 (TAG) and 698 (TAG), respectively.

All the above frameshift and nonsense mutations generating premature termination of translation and thus a loss-of-function of MMR, and contributing to the neoplastic transformation process are thought to be pathogenic mutations^14^.

Two splice site mutations were detected: 1 in *MSH6* and 1 in *MSH2*. The mutation in *MSH6* was c.3557-2 A > G adjacent to exon 7, which had not previously been reported. Variants at the same site (c.3557-2 A > T) were recorded as “likely pathogenic” in the ClinVar database (http://www.ncbi.nlm.nih.gov/). The *MSH2* splice site mutation was due to c.2211-2A > G adjacent to exon 14, which was recorded as “pathogenic/likely pathogenic” in the ClinVar database (http://www.ncbi.nlm.nih.gov/ (May 17, 2018)). Splice site mutations can interfere with splicing and cause abnormally spliced mRNA transcripts, thus leading to synthesis of nonfunctional MMR proteins.

One missense mutation, c.2041G > A p.Ala681Thr, was found in *MLH1* and was considered a “pathogenic” missense mutation by InSiGHT (Dec 18, 2013) and Invitae (Sep 11, 2015) in the ClinVar database (http://www.ncbi.nlm.nih.gov/). The mutation has been reported in the Chinese population^15^.

### MSI analysis

**Figure 2:**
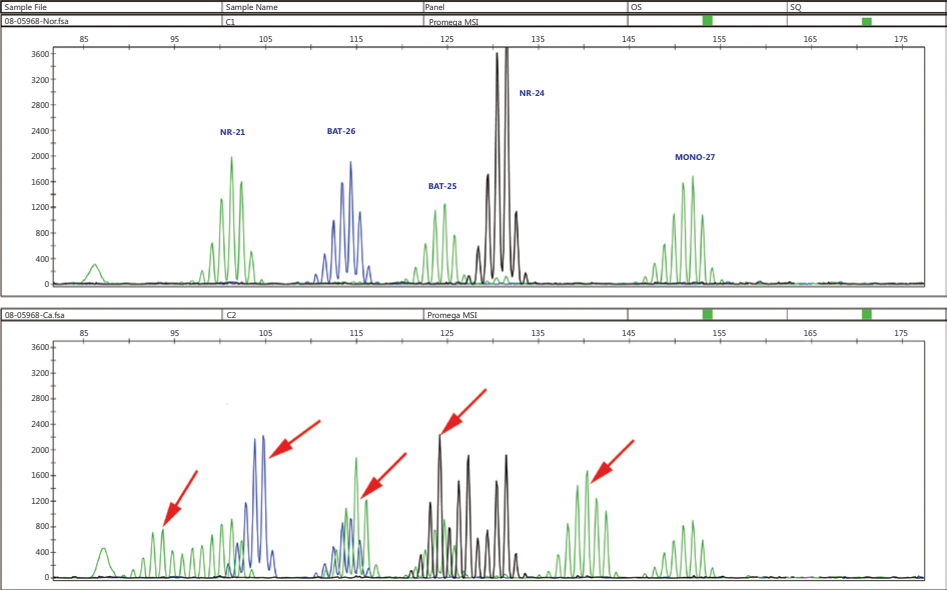
MSI analysis in MMR mutation tumors. Top panel: normal control; Bottom panel: MSI-positive samples. The presence of new alleles in the test sample (indicated by arrows) indicates MSI.

For cases in which MMR mutations were detected by NGS, we performed MSI testing to verify the function of these variants. We found that 100% of tumors (10/10) were MSI-H. Most of the samples (70%, 7/10) were unstable at 3 markers, whereas 1 showed instability at 4 markers, and 2 showed instability at 5 markers, thus indicating that these variants are pathogenic mutations (**Figure 2**).

## Discussion

Currently, the diagnosis of LS requires documentation of at least 1 MMR gene mutation. However, universal sequencing of all endometrial carcinoma cases is not a cost-effective option, given that less than 5% of EC cases are LS associated^16,17^. Therefore, a strategy that maximizes clinical benefit and minimizes costs is urgently needed.

To date, IHC and/or MSI with MLH1 methylation analysis have been recommended to identify suspected LS cases. However, simultaneous screening with IHC and MSI may be neither essential nor cost-effective, and compliance with the screening guidelines is low in the United States. Molecular MSI testing cannot specify which MMR gene is involved^18,19^. In addition, IHC results are highly consistent with the MSI-H phenotype in newly diagnosed patients with EC and CRC^20^. In a comparative study on CRC samples from our center, IHC showed 100% (245/245) specificity and 99.1% (243/245) sensitivity, as compared with MSI. The only 2 inconsistent cases that were positive by IHC and MSI-H were finally found to be sporadic cases due to MLH1 methylation^21^. Therefore, we chose IHC as the first step in our LS screening procedure, then excluded sporadic EECs by MLH1 methylation testing, and ultimately confirmed the LS cases through both NGS and MSI analysis. We found that 10 EEC (10/209) cases had MMR variants, all of which were MSI-H, thus indicating that IHC is highly consistent with MSI. In addition, among these cases, 7 had truncated proteins, 2 had abnormal splicing, and 1 had a pathogenic missense mutation, all of which resulted in MSI-H. These mutations were not found in the 5000 Exomes database (population frequency information from the 5000 Exomes Project) and hence were actual pathogenic mutations. Among these mutations, 3 previously unreported variants were detected in our study.

The biallelic deletion in *MSH6* (c.597_597delC) was found in 4 unrelated patients without known consanguinity, who lived in different provinces of China. Each case with this mutation was associated with a wide spectrum of cancer family history, such as CRC, cervical adenocarcinoma, liver cancer, or lung cancer. One patient developed CRC 10 years after EEC onset. In addition, 3 family members had appendicitis several years before the onset of EEC. Family history is very helpful in LS screening and diagnosis. The first family with LS (Family G), found by Warthin in 1895, was reported to present with various types of cancers, and 3 family members also had appendicitis^22^. Whether appendicitis is associated with the onset of LS is unknown and remains to be confirmed in multiple cases. Traditional methods to identify patients with LS, such as the Amsterdam II criteria and revised Bethesda Guidelines, are based on family histories of cancer^23–25^. Unfortunately, family history is not always available or reliable. In present-day China, pedigrees are increasingly unavailable because of the nationwide predominance of small families as a result of family planning in the past several decades. Therefore, combining family history and genetic testing is preferable to confirm LS. The functional consequences of this deletion mutation were found to be a truncated protein and thus a loss-of-function of MMR and MSI-H, genome instability, and increased susceptibility to various tumors. The high rate of this mutation in the Chinese population indicated that it is probably a founder mutation in China. A large-scale population-based study is needed to further analyze the presence of this founder mutation in the Chinese population with LS.

Ten patients with EEC (10/209, 4.78%) were pathogenic mutation carriers. Because of large discrepancies in ethnicities, ages, sample restrictions, MSI selection, sequencing methods, and mutation evaluation processes, a wide range of *MMR* mutation rates have been reported among different EC cohorts (1.8% in the USA, 2.1% in Finland, 4.6% in Spain, and 8.3% in Japan)^5,26–28^. In the current study, *MSH6* mutations were the most prevalent (70%, 7/10) and represented 3.35% (7/209) of our EEC series—findings comparable to published results. Among 543 endometrial carcinoma samples detected by Hampel et al., *MSH6* mutations were the most common (0.92%, 5/543), followed by *MSH2* mutations (0.37%, 2/543) and *MLH1* mutations (0.18%, 1/543)^7^. Buchanan et al. have reported similar results and have found that most mutation carriers (17 of 22) have endometrioid subtype cancer^12^. Devlin et al. have demonstrated that *MSH6* mutations are more common in patients with endometrial carcinoma than in families with CRC (3.8% *vs.* 2.6% of truncating mutation)^29^. Baglietto et al. have further emphasized the uniqueness of the* MSH6* mutation in endometrial carcinoma: women carrying *MSH6* mutations are more prone to developing EC than CRC: the EC morbidity is 26 times higher for *MSH6* mutation carriers, whereas the incidence of CRC is only 8-fold higher, and the incidence of other LS-associated cancers is only 6-fold higher^3^.

Similarly to the findings in recent studies^30,31^, 37.5% (6/16) of our unmethylated MMR-deficient EC patients did not have a pathogenic germline mutation. Thus, the cases fell into the “Lynch syndrome-like” category. Some cases were characterized by somatic biallelic inactivation, and some are likely to be true LS cases with germline mutations that could not be detected, possibly because of (1) mutations in untested regulatory regions of MMR genes, such as the promoter regions, untranslated regions, or deep intron sequences^32^; (2) genomic rearrangements or complex mutations that are not readily identified by current sequencing techniques; or (3) direct or indirect involvement of MMR function via germline mutations in other genes, such as EPCAM, MLH3, MSH3, EXO1, PMS1, and TGFBR. Of note, the main limitation of this study is that it is based on data from only a single institution. Therefore, multi-center research is needed for further confirmation of the results. A study on a larger population and including samples from other institutions is ongoing.

## Conclusions

Overall, we identified 3 previously unreported mutations and detected a probable founder mutation from a Chinese EEC population. We also demonstrated a universal screening strategy for LS testing in Chinese patients with EEC through IHC for primary screening, followed by *MLH1* methylation analysis, and final confirmation by NGS and MSI testing. We anticipate that the mutations found in this study will expand understanding of LS and that the strategy we established will be helpful as a rapid and reliable method to LS screening in patients with EEC.

## Figures and Tables

**Table 1:** Patients involved in the present investigation

Variables	MMR-retained cases (*n*)	MMR-deficient cases (*n*, %)	Total cases	Mean age, years (range)
Endometrial endometrioid cancer (EEC)	184	27 (12.80)	211	54.64 (25–80)

**Table 2:** IHC patterns with suspected LS in EEC

IHC patterns	Suspected LS *n* (%)	Mean age (years)	*P*
EEC	27	56.22	
Loss of MLH1	13 (13/27 = 48.15%)	60	0.24
Loss of MSH2/MSH6	10 (10/27 = 37.04%)	51. 3	
Loss of MSH6 alone	4 (4/27 = 14.81%)	56.25	

**Table 3:** Germline MMR mutations in different patients

Cases	Age	IHC loss	MMR gene	Exon	Nucleotide	Consequence
EEC001	50	MSH2/MSH6	*MSH6*	3	c.595_595delC	p.Ser200fs
EEC002	60	MSH2/MSH6	*MSH6*	3	c.595_595delC	p.Ser200fs
EEC003	66	MSH2/MSH6	*MSH6*	3	c.595_595delC	p.Ser200fs
EEC004	49	MSH2/MSH6	*MSH6*	3	c.595_595delC	p.Ser200fs
EEC005	58	MSH2/MSH6	*MSH6*	1	c.71C > A	p.Ser24Ter
EEC006	55	MSH2/MSH6	*MSH6*	9	c.3906_3907insGCAAG	p.Leu1305fs|
EEC007	53	MSH2/MSH6	*MSH6*	7	c.3557-2 A > G	
EEC008	42	MSH2/MSH6	*MSH2*	14	c.2211-2A > G	
EEC009	31	MSH2/MSH6	*MSH2*	13	c.2092G > T	p.Glu698Ter
EEC010	58	MLH1	*MLH1*	18	c.2041G > A	p.Ala681Thr
Mean	52.2					

**Table 4:** Germline mutation types in MMR genes

Mutation types	MMR gene	Exon	Nucleotide	Consequence	Codon	Function	Cases
Frameshift deletion	*MSH6*	3	c.595_595delC	p.Ser200fs	TAG	Pathogenic	4
	*MSH6*	9	c.3906_3907insGCAAG	p.Leu1305fs	TGA	Pathogenic	1
Nonsense	*MSH6*	1	c.71C > A	p.Ser24Ter	TAG	Pathogenic	1
	*MSH2*	13	c.2092G > T	p.Glu698Ter	TAG	Pathogenic	1
Splice site	*MSH2*	14	c.2211-2A > G			Pathogenic/likely pathogenic	1
	*MSH6*	7	c.3557-2 A > G			Likely pathogenic	1
Missense	*MLH1*	18	c.2041G > A	p.Ala681Thr	ACT	Pathogenic	1

**Table 5:** Clinical features of cases with MSH6 c.595_595delC from 4 unrelated cases

Cases	Age	Diagnosis	FIGO stage	Gradea	Family history/personal history
E001	50	EEC	II	G2	Acute appendicitis (herself, 13 years ago); cervical adenocarcinoma (mother); CRC (brother, uncle); lung cancer (brother); liver cancer (uncle)
E002	60	EEC	Ia	G2	Chronic appendicitis (herself, 27 years ago); CRC (herself, 10 years later); CRC (mother)
E003	66	EEC	II	G2	Cervical adenocarcinoma (mother); CRC (brother)
E004	49	EEC	Ib	G3	Chronic appendicitis (6 years ago); CRC (mother)

^a^G1: high differentiation; G2: moderate differentiation; G3: poor differentiation.
