# Supplementary material for: Identification of novel Lynch syndrome mutations in Chinese patients with endometriod endometrial cancer
Source: Cancer Biol Med. 2020 May 15;17(2):458–67. doi: 10.20892/j.issn.2095-3941.2019.0295 (PMC7309470; doi:10.20892/j.issn.2095-3941.2019.0295)
Supplement: Supplementary file 1 [file cbm-17-458-s001.pdf]

Supplementary materials

Details of the prediction methods

PolyPhen-2 (http://genetics.bwh.harvard.edu/pph2/) scores ranged from 0.0 (tolerated) to 1.0 (deleterious). Variants with scores of 0.0 were predicted to be benign, and those with scores closer to 1.0 were more confidently predicted to be deleterious. The score can be interpreted as follows: variants with scores in the 0.0–0.15 range were predicted to be benign; variants with scores in the 0.15–1.0 range were possibly damaging; and variants with scores in the 0.85–1.0 range were confidently predicted to be damaging. Sift (http://sift.jcvi.org/) and PolyPhen-2 scores used the same range of 0.0–1.0 but had opposite meanings: variants with scores in the 0.0–0.05 range were considered deleterious; variants with scores closer to 0.0 were more confidently predicted to be deleterious; variants with scores in the 0.05–1.0 range were predicted to be tolerated (benign); and variants with scores very close to 1.0 were confidently predicted to be tolerated.

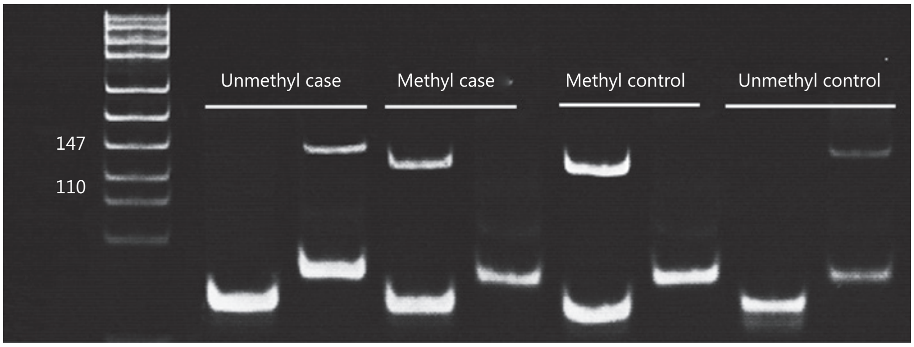

Figure S1 Methylation testing for MLH1 in MLH1 loss cases. Lane 1, 2: unmethylated MLH1 in EEC sample; Lane 3, 4: methylated MLH1 in EEC sample; Lane 5, 6: methylated MLH1 positive control; Lane 7, 8: unmmethylated MLH1 control.

Table S1 Clinicopathologic features by MMR expression in EEC

Variables	MMR-retained cases, *n* (%)	MMR-deficient cases, *n* (%)	*P*
EEC	184 (87.20)	27 (12.80)	
Age (median)			
54.64 (25–80)	54.39 (25–80)	56.22 (31–76)	
<50	56 (30.43)	6 (22.22)	0.382
≥50	128 (69.57)	21 (77.78)	
Stage at initial diagnosis	141	18	
I/II	127 (90.07)	17 (94.44)	0.55
III/IV	14 (9.93)	1 (5.56)	
Grade (FIGO)	181	25	
1/2	163 (90.06)	24 (96)	0.336
3	18 (9.94)	1 (4)	
Depth of myometrial invasion	151	21	
Superficial (<1/2)	109 (72.19)	15 (71.43)	0.942
Deep (≥1/2)	42 (27.81)	6 (28.57)	
Low uterus segment	131	27	0.137
No	113 (86.26)	21 (77.78)	
Yes	18 (13.74)	6 (22.22)	
Lymphovascular invasion	155	22	
No	141 (90.97)	21 (95.45)	0.479
Yes	14 (9.03)	1 (4.55)	
Tumor infiltrating lymphocytes			
No	35 (23.18)	2 (9.1)	0.28
Yes	116 (76.82)	20 (90.9)	
Peritumoral lymphocytes			
No	118 (85.51)	9 (81.82)	0.74
Yes	20 (14.49)	2 (18.18)	

Table S2 Features of MLH methylation in EEC

Variables	Methylated MLH1, *n* (%)	Unmethylated MLH1, *n* (%)	*P*
	13 (76.47)	4 (23.53)	
Age (median)	56.46 (32–76)	55.75 (49–63)	0.856
Stage at initial diagnosis			
I/II	9 (90)	3 (100)	0.57
III/IV	1 (10)	0 (0)	
Grade (FIGO)			
1/2	11 (91.67)	4 (100)	0.55
3	1 (8.33)	0 (0)	
Depth of myometrial invasion			
Superficial (<1/2)	10 (81.82)	3 (75)	0.423
Deep (≥1/2)	1 (18.18)	1 (25)	
Lymphovascular invasion			
No	10 (81.82)	3 (75)	0.423
Yes	1 (18.18)	1(25)	
Low uterus segment			
No	6 (60)	3 (75)	0.6
Yes	4 (40)	1 (25)	
Tumor infiltrating lymphocytes			
No	6 (60)	3 (75)	0.6
Yes	4 (40)	1 (25)	
Peritumoral lymphocytes			
No	5 (71.43)	3 (75)	0.9
Yes	2 (28.57)	1 (25)	
